# The Scientist Who Described the Filarial Parasite *Mansonella ozzardi* in 1897

**DOI:** 10.3201/eid2807.210818

**Published:** 2022-07

**Authors:** James Lee Crainey, Uziel Ferreira Suwa, Sérgio Luiz Bessa Luz

**Affiliations:** Fundação Oswaldo Cruz Amazônia, Manaus, Brazil

**Keywords:** Mansonella ozzardi, Filaria Demarquayi, filariae, nematodes, parasites, Sir Patrick Manson, Dr. Albert Tronson Ozzard

## Who is this person?

Here is a clue: He first described the filarial parasite *Mansonella ozzardi* in 1897.

Sir Patrick Manson

B) George Carmichael Low

C) Albert Tronson Ozzard

D) Joseph Bancroft

E) Thomas Lane Bancroft

Decide first. Then turn the page.

## Sir Patrick Manson

Working in the port of Amoy (now known as the port of Xiamen), China (*1*–*5*), Sir Patrick Manson (Figure) noted that *Wuchereria bancrofti* microfilariae appeared with marked periodicity in the blood of infected persons and that they developed inside blood-fed mosquitoes (*1*–*4*) (https://era.ed.ac.uk/bitstream/handle/1842/28457/LowGC_1910redux.pdf). Although Manson did not initially think that mosquito bites were the mode by which these parasites were transmitted, his study was revolutionary because it was the first to show that insects play a role in infectious disease transmission (*1*–*4*) (https://era.ed.ac.uk/bitstream/handle/1842/28457/LowGC_1910redux.pdf). In the ≈150 years since Manson’s observation, millions of lives and disability-adjusted life-years have been saved by interventions developed to control vectorborne diseases, nearly all of which can in some way or another trace their provenance to his discovery (*5*). Manson’s effect on the field of global health research is, however, by no means limited to the legacy of his research discoveries and by no means entirely uncontroversial (*1*–*3*,*6*).

**Figure:**
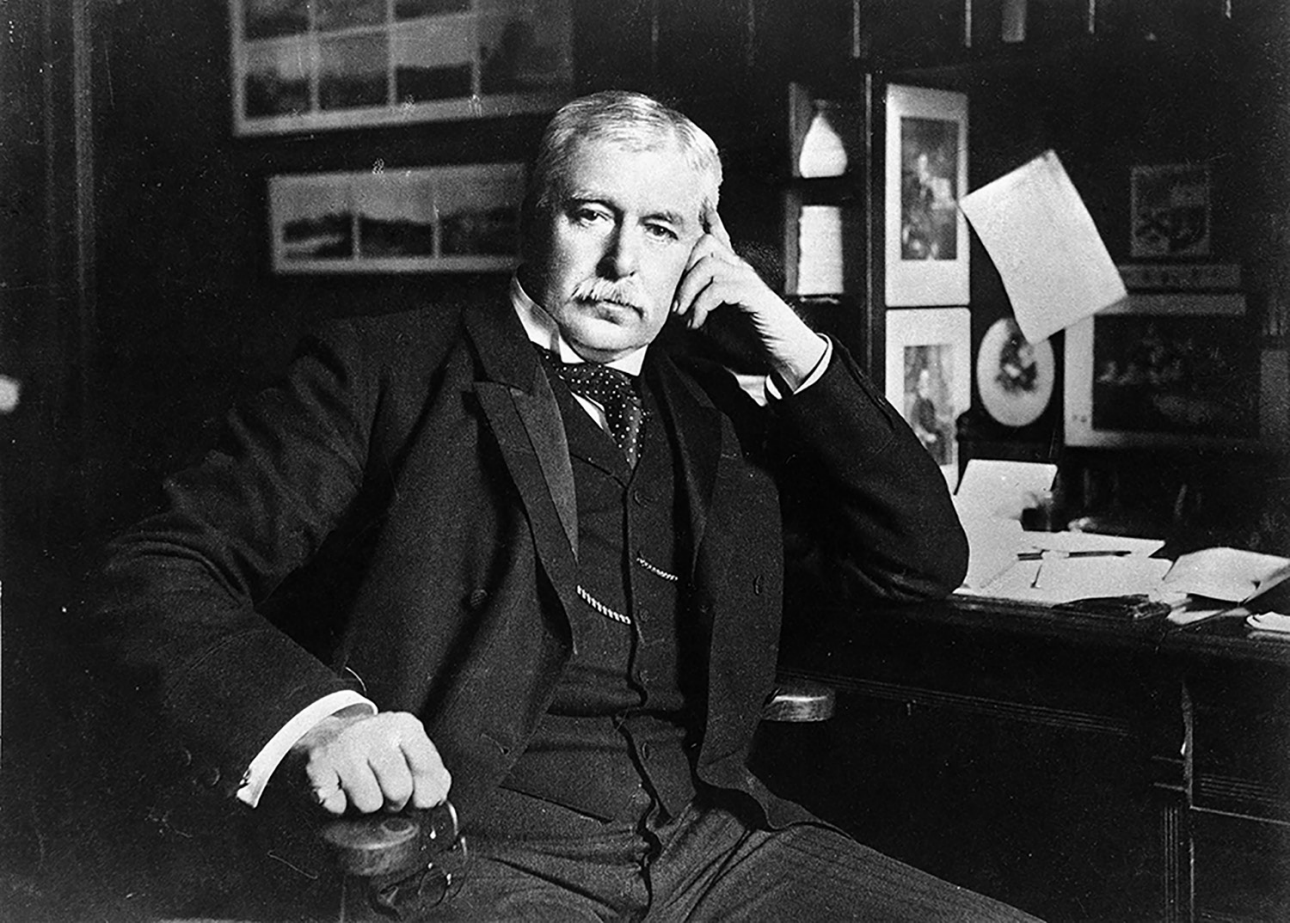
Sir Patrick Manson

At the International Congress of Medicine, held in London in August 1913, Sir Patrick Manson was awarded a gold medal and commemorative plaque in recognition of his contributions to the field of tropical medicine (*1*). As French Professor Raphaël Blanchard, who had the medal designed and struck, presented the awards, he described Manson as the “father of tropical medicine” (*1*). Although global health researchers today increasingly recognize that Manson was deeply complicit in the colonization of global health, there are still many good reasons why Blanchard’s “father of tropical medicine” sobriquet has managed to stick (*1*–*3*,*6*).

In addition to his role in creating medical schools and societies in Hong Kong, where he lived and worked early in his professional career, Manson successfully persuaded his own national government of its need to invest in tropical disease research and training infrastructure within Britain for the health and prosperity of its wider empire (*1*–*3*,*6*). As a medical adviser to the colonial office, Manson successfully advocated for creating the Royal Society of Tropical Medicine (RSTM) and the foundation of the London School of Hygiene and Tropical Medicine (LSHTM) and the Liverpool School of Tropical Medicine (LSTM), which both remain in operation to this day (*1*–*3*,*6*). In fact, the LSTM and LSTHM are now widely regarded as being among the world’s most prestigious tropical medicine research institutes. Although the RSTM does not command the same level of authority it once did, it still exerts influence over the field of tropical disease research through its various house journals, grant schemes, prizes, and prominent presidents (https://rstmh.org) (*1*–*3*,*6*).

Through teaching and mentoring staff and students inside and outside of these institutes, Manson also effected the direction of scientific inquiry that followed his own (*1*–*3*). By showing that mosquitoes can inoculate people with malaria and filarial parasites, Sir Ronald Ross, George Carmichael Low, and Thomas Lane Bancroft played critical roles in advancing Manson’s vector research (*1*–*3*,*6*). Although these men are now all regarded as accomplished scientists in their own right, there is no doubt that all 3 benefited greatly from Manson’s mentorship and support (*1*–*3*,*6*). Manson’s guide to tropical medicine, which he wrote and updated 6 times during his lifetime, is arguably, however, a more valuable and lasting contribution to research training than was his mentoring. First published in 1897, and then for over a century edited by some of the field’s most eminent research scientists, *Manson’s Tropical Disease* is now in its 23rd edition and continues to be among the most authoritative tropical medicine reference textbooks for students, researchers, and practitioners (*7*).

This book title is, however, not the only surviving tribute to Manson’s name; several parasites, disease vectors, and diseases are named after him (*7*–*9*). In homage to Manson, when Ernest Carroll Faust created the *Mansonella* genus in 1929 and classified *M. ozzardi* within it, he also proposed that the human disease it causes should be named mansonelliasis (*8*). After a series of taxonomic revisions in the 1980s, 2 more filarial parasites of humans (*M. perstans* and *M. streptocerca*) were placed within the *Mansonella* genus; thus, today the terms mansonelliasis and mansonellosis are both commonly used to describe the microfilaremic disease caused by any of these 3 filarial parasites (*9*).

At Blachard’s suggestion, when Manson first described *M. ozzardi*, he decided to name it after the French surgeon Jean Nicolas Demarquay (*10*). This naming seemed a fitting tribute for Manson to make to the man who first discovered the parasite he had used to make his name (*Wuchereria bancrofti*) but whose discovery was not initially recognized by the international research community (*1*,*2*) (https://era.ed.ac.uk/bitstream/handle/1842/28457/LowGC_1910redux.pdf). Manson’s homage would, however, not endure because when he first described *M. ozzardi*, he used the name *Filaria demarquayi* to describe parasites from the Caribbean and the name *Filaria ozzardi* to describe parasites from Guyana (*10*). Although the question of whether parasites from the Caribbean and *M. ozzardi* parasites from mainland South America should be regarded as separate species remained controversial until their DNA was comparted more than a century later (*11*), Manson would eventually conclude (correctly) that both were of the same species and that *F. demarquayi* should be treated as a synonym for *F. ozzardi* (*12*).

Manson’s decision to synonymize *F. demarquayi* was surprising because he had made clear in his original description of the species that he had found Caribbean *F. demarquayi* parasites several years before he had seen the Guyana parasites and because a report of Manson’s discovery of *F. demarquayi* parasites had also been published in the Proceedings of the Academy of Natural Sciences of Philadelphia before his British Medical Journal publication (*12*,*13*). The name *F. demarquayi* thus seemed to have primacy by most standard nomenclature protocols. However, when erecting the *Mansonella* genus, Faust followed Manson’s synonymization proposal, arguing that the *F. demarquayi* species name had already been used to describe a dubious filarial parasite species in 1892 and was thus unavailable as a name for Manson’s parasites (*8*).

The contemporary name for the parasite species, *Mansonella ozzardi*, thus pays homage to Manson and to an industrious colonial medical officer from present-day Guyana, Albert Tronson Ozzard, who had only a limited role in the parasite’s description (*8*,*10*,*14*). With modern researchers and research institutions, including those that Manson founded, increasingly recognizing the need for the decolonization of global health, it is thus tempting to suggest that changing the name of a New World parasite species, whose contemporary name pays homage to 2 British colonial servants and does not obey conventional nomenclature protocols, could contribute to such a process (*6*,*8*,*14*). The distribution of mansonellosis seems to be restricted to poor regions of Latin America, the Caribbean, and Africa, where Manson never set foot (*3*,*9*). Given all this and the fact that *M. perstans*, like onchocerciasis, most likely arrived in Latin America because of slavery, an argument can thus be made that changing the name of mansonellosis could also contribute to the decolonization process (*15*).

Presently, there are no specific disease burden estimates for mansonellosis, and only mild symptoms (e.g., joint pains, chills, headaches, corneal lesions) have been robustly linked to it (*9*). Perhaps after more is known about mansonellosis, it will be time for it to have a name that reflects its public health burden rather than a name that pays tribute to the colonial father of tropical disease, Sir Patrick Manson.
